# UroMark—a urinary biomarker assay for the detection of bladder cancer

**DOI:** 10.1186/s13148-016-0303-5

**Published:** 2017-01-31

**Authors:** Andrew Feber, Pawan Dhami, Liqin Dong, Patricia de Winter, Wei Shen Tan, Mónica Martínez-Fernández, Dirk S. Paul, Antony Hynes-Allen, Sheida Rezaee, Pratik Gurung, Simon Rodney, Ahmed Mehmood, Felipe Villacampa, Federico de la Rosa, Charles Jameson, Kar Keung Cheng, Maurice P. Zeegers, Richard T. Bryan, Nicholas D. James, Jesus M. Paramio, Alex Freeman, Stephan Beck, John D. Kelly

**Affiliations:** 1 UCL Cancer Institute, University College London, London, UK; 2 Division of Surgery & Interventional Science, UCL Medical School, University College London, London, UK; 3 Molecular Oncology Unit, CIEMAT (ed70A), Madrid, Spain & Biomedical Research Institute I+12, Universitary Hospistal 12 de Octubre, Av Cordoba s/n. 28041, Madrid, Spain; 4 Department of Histopathology, University College London Hospital, London, UK; 5 School of Cancer Sciences, University of Birmingham, Birmingham, UK; 6 Institute of Applied Health Research, University of Birmingham, Birmingham, UK; 7 School for Public Health and Primary Care, Maastricht University, Maastricht, Netherlands; 8 Cancer Research Unit, University of Warwick, Coventry, UK; 9 Uro-oncology Section & Biomedical Research Institute I+12, Universitary Hospital 12 de Octubre, Av Córdoba s/n. 28041, Madrid, Spain; 10 Centro de Investigación, Biomédica en Red de Cáncer (CIBER ONC), Madrid, Spain

**Keywords:** Bladder cancer, Epigenetics, Urine, Next generation sequencing, Diagnostic

## Abstract

**Background:**

Bladder cancer (BC) is one of the most common cancers in the western world and ranks as the most expensive to manage, due to the need for cystoscopic examination. BC shows frequent changes in DNA methylation, and several studies have shown the potential utility of urinary biomarkers by detecting epigenetic alterations in voided urine. The aim of this study is to develop a targeted bisulfite next-generation sequencing assay to diagnose BC from urine with high sensitivity and specificity.

**Results:**

We defined a 150 CpG loci biomarker panel from a cohort of 86 muscle-invasive bladder cancers and 30 normal urothelium. Based on this panel, we developed the UroMark assay, a next-generation bisulphite sequencing assay and analysis pipeline for the detection of bladder cancer from urinary sediment DNA. The 150 loci UroMark assay was validated in an independent cohort (*n* = 274, non-cancer (*n* = 167) and bladder cancer (*n* = 107)) voided urine samples with an AUC of 97%. The UroMark classifier sensitivity of 98%, specificity of 97% and NPV of 97% for the detection of primary BC was compared to non-BC urine.

**Conclusions:**

Epigenetic urinary biomarkers for detection of BC have the potential to revolutionise the management of this disease. In this proof of concept study, we show the development and utility of a novel high-throughput, next-generation sequencing-based biomarker for the detection of BC-specific epigenetic alterations in urine.

**Electronic supplementary material:**

The online version of this article (doi:10.1186/s13148-016-0303-5) contains supplementary material, which is available to authorized users.

## Background

Bladder cancer represents one of the most common malignancies in the western world, ranking 8th in incidence and ranks 13th in terms of cancer mortality worldwide [1]. Cystoscopy is the gold standard test for the detection of bladder cancer, but it is operator-dependent with a sensitivity of 90–97% [2–6]. However, cystoscopy is an invasive procedure requiring clinic or hospital attendance and poses a small but significant risk of infection [7]. Although the sensitivity of cystoscopy is less than absolute, patient perception of the test is such that the performance of an alternative (such as a non-invasive) test should have a sensitivity of 95% or greater [8].

Bladder cancer carries a significant health economic burden in the UK, with the management of bladder cancer costing in excess of £55M/year [9, 10]. A significant proportion of that cost is due to the need for cystoscopy to rule out the presence of cancer. Over 110,000 cystoscopies are performed each year in the UK for patients presenting with haematuria, and a similar number for surveillance cystoscopies are performed for known non-muscle-invasive bladder cancer patients. However, given that only 10% of haematuria patients undergoing cystoscopy will have a diagnosis of bladder cancer, a non-invasive assay which can rule out the presence of cancer with a high degree of certainty will not only reduce the economic burden of cystoscopy, but also minimise the requirement for this invasive procedure in the majority of patients without cancer [9, 10]. Although several commercial assays have FDA approval for the detection of bladder cancer from urine, none are approved as stand-alone tests to replace cystoscopy [11]. This includes urine cytology, which is frequently used as a diagnostic aid in conjunction with cystoscopy, but as with the other commercial assays, has low sensitivity to detect cancer other than high-grade disease, and carcinoma in situ thus cannot replace cystoscopy [12, 13].

Changes in DNA methylation play a key role in malignant transformation, leading to the silencing of tumour suppressor genes and overexpression of oncogenes [14]. Despite its plasticity, DNA methylation is ontogenically relatively stable, a property which can be exploited to develop diagnostic assays resulting in an active area of research in the field of urinary-based biomarkers for the non-invasive detection of bladder cancer [15–18]. To date, DNA methylation biomarker panels have contained a relatively small number of loci, in part due to technological limitations and the requirement to retain diagnostic specificity [11–19]. Although these panels have shown promise [19], they have in general not reached the required sensitivity to replace cystoscopy with an inherent weakness being the limited number of targets included to maintain specificity [19–24]. More recently, assays based on mutation and methylation targets have shown high sensitivity but remain to be validated [20, 25].

Emerging techniques that utilise next-generation DNA sequencing (NGS) hold particular promise for the development of highly sensitive epigenetic biomarker panels. For example, the microdroplet-based PCR amplification of bisulfite-converted DNA followed by NGS of the amplified target loci (termed RainDrop BS-Seq) enables the sensitive, specific and simultaneous amplification of up to 4000 bisulfite-converted target loci [26]. We and others have shown the utility of this approach to validate epigenetic alterations in a range of tissues [27–29]. In this proof of concept study, we describe the development of the UroMark assay, which uses high-throughput targeted bisulphite sequencing of urinary sediment cell DNA to provide a read-out of presence or absence of bladder cancer. This assay, in which a large comprehensive panel shows diagnostic precision, achieving high sensitivity and specificity for disease detection, would represent a potential paradigm shift in the diagnosis and surveillance of bladder cancer.

## Methods

### Study population

Genome-wide DNA methylation profiling was performed on DNA from 86 bladder cancers and 30 age-matched normal urothelium samples obtained from biorepositories at the University College London Hospitals (UCLH) and the University of Birmingham Bladder Cancer Prognosis Program (BCPP) (cohort 1, Table 1). Pathological review of representative haematoxylin and eosin (H&E) sections was conducted to include only specimens with tumour cellularity >80%. Blood methylome data was retrieved from the MARMAL-aid database (http://marmal-aid.org, [30]). Normal urothelium samples were taken from non-bladder cancer patients by urothelial brushings to ensure a true reflection of the normal urothelium and limit contamination from underlying stroma and muscle.

**Table 1:** Patient characteristics of primary tissues used in (cohort 1) discovery and (cohort 2) validation

	Cohort 1 (*N* = 116)	Cohort 2 (*N* = 199)
Cancer	86	179
Age	68 (32–90)	68 (34–88)
Gender		
Male/Female	52/34	132/47
Ta–T1	16	35
T2–T4	70	144
Low grade	12	35
High grade	74	144
Non-cancer	30	20
Age	62 (45–86)	67 (41–82)
Gender		
Male/Female	22/8	23/7

For target validation, cohort 2 (*n* = 199) was an independent dataset obtained from The Cancer Genome Atlas (TCGA) (https://tcga-data.nci.nih.gov/tcga/dataAccessMatrix.htm?mode=ApplyFilter&showMatrix=true&diseaseType=BLCA&tumorNormal=TN&tumorNormal=T&tumorNormal=NT&platformType=2&platformType=42). This contains data for 144 muscle-invasive bladder cancers and 20 normal urothelium samples. We supplemented the TCGA data with a further 35 methylomes generated from non-muscle-invasive disease, representative of low-grade disease from the Centro de Investigaciones Energéticas, Medioambientales y Tecnológicas (CIEMAT) (Madrid) (cohort 2, Table 1).

Cohorts 3 and 4 comprised voided urine samples obtained from the patients attending the UCLH for investigation of haematuria and surveillance cystoscopy. Haematuria cases were investigated by cystoscopy and upper tract imaging as standard of care [31]. A visual diagnosis of cancer was confirmed by tumour resection and histopathological analysis. Voided urine samples were obtained between January 2012 and 2016 (cohorts 3 and 4 (Table 2)), and the urinary sediment was pelleted and stored at −80 °C. Cohort 3 (*n* = 86) comprised 52 confirmed bladder cancer and 34 non-bladder cancer cases. Cohort 4 (*n* = 205) comprised 55 bladder cancer and 133 non-bladder cancer cases. The cellular content of urine samples was pelleted by centrifugation at 1500*g* for 10 min and the supernatant removed. Urinary DNA was extracted using a DNeasy Blood and Tissue Kit (Qiagen). The cell pellet was washed with PBS and repelleted, and the supernatant was removed and the pellet resuspended in 200 μL fresh PBS. The samples were digested with Proteinase K and incubated at 56 °C for 10 min, and 200 μL of absolute ethanol was added before transfer to DNeasy columns. DNA was extracted according to the manufacturer’s instructions and finally eluted in 100 μL of Buffer AE (Qiagen). The DNA was quantified by spectrophotometry (Nanodrop 1000) and fluorimetry (Qubit dsDNA HS Assay Kit, Invitrogen). The DNA integrity was assessed using a Bioanalyzer (Agilent Technologies).

**Table 2:** Patient characteristics of urine samples used in the assessment of the UroMark assay

	Cohort 3 (*N* = 86)	Cohort 4 (*N* = 188)
Cancer	52	55
Age	62.4 (22–111)	65.2 (36–90)
Gender		
Male/Female	49/13	43/12
Ta–T1	27	28
T2–T4	25	27
Low grade	17	24
High grade	35	31
Non-cancer	34	133
Age	62 (27–89)	63 (29–144)
Gender		
Male/Female	20/14	82/51
Haematuria status		
Micro	4	67
Macro	11	51
Unknown	19	15

### Ethics approval

The studies were conducted under the following ethics approvals: For primary tissue obtained at UCLH (10/H1306/42, 15/YH0311), BCPP (06/MRE04/65) and Madrid (CEIC 10/50); and urinary validation: 06/Q0104/57, 10/H1306/42 and 15/YH0311.

### Genome-wide methylation profiling

Five hundred nanograms of DNA was bisulfite-converted and hybridised to the Infinium 450K Human Methylation array (Illumina) and processed in accordance with the manufacturer’s recommendations. DNA bisulfite conversion was carried out using the EZ DNA Methylation Kit (Zymo Research) as per manufacturer’s instructions. The *R* statistical software (version 3.1.2 [32]) was used for the subsequent data analysis. The ChAMP analysis pipeline was used to extract and analyse data from iDat files [33]. Samples were normalised using BMIQ [33, 34]. Raw *β*-values (methylation value) were subjected to a stringent quality control analysis as follows: samples showing reduced coverage and probes containing SNPs were removed, and only probes with detection levels above background across all samples were retained (detection *P* < 0.01).

### Panel identification, selection and classification

The UroMark panel was defined using pre-set criteria in the training cohort (Table 1) as follows: in order for probes to be considered as potential biomarker candidates, they had to show no or very low methylation (*β* <10%) in normal urothelium, blood and non-cancer urine samples and methylation (*β*) of >50% in bladder cancer. A <10% cut was selected from the analysis of beta values from fully unmethylated control DNA. The probes passing this filter were subsequently used to generate a classifier using a random forest model.

The random forest classification model was selected as it has been shown to be effective with a limited number of predictors (i.e. number of loci being compared) in comparison with the number of training points (i.e. the number of samples within the training cohort) [35]. The random forest model was implemented through the Bioconductor package CAReT (version 6.0-24) [35]. To define a robust classifier, the following steps were implemented: (A) A random selection of 80% of cases and controls was selected as training cohort, with the remaining 20% retained to form a test set; (B) a random forest model was developed for each training cohort; (C) a predicted class (cancer or normal) was generated for each corresponding test cohort; (D) for each iteration, the model and area under the curve (AUC) value were noted; (E) steps A–D were repeated 100 times, and the optimal model was determined by comparison of each AUC value. The optimal model was fixed and applied to all subsequent analysis.

Comparative testing of UroMark assay and classifiers based on the best performing 3, 5 and 10 loci panels was performed by a simple logistic regression model to calculate probabilities of each combination. The performance of individual loci is shown in Additional file 1: Table S4. The best performing combination of 3, 5 and 10 probes were selected based on a false positive rate of <10% in the training cohort. Area under the ROC (receiver operating characteristic) curve for the UroMark assay and best performing 3, 5 and 10 marker panels were calculated using the Bioconductor package pROC (version 1.7). Loci were involved in *OTX1*, *ONECUT2*, *ZNF154*, *TBX2* and *ZIC4*.

### RainDance microdroplet PCR of urinary DNA

RainDrop BS-seq was performed as previously described [27, 28]. Primers were designed for targeted regions in Additional file 2: Table S1. For microdroplet PCR, 7.20 μL of bisulfite-treated urinary DNA was added to 4.70 μL of 10× High-Fidelity Buffer (Invitrogen), 1.80 μL of 50 mM MgSO_4_ (Invitrogen), 1.62 μL of 10 mM dNTP solution mix (NEB), 3.60 μL of 4 mol L^−1^ betaine solution (Sigma-Aldrich), 3.60 μL of droplet stabiliser (RainDance Technologies), 1.80 μL of 100% dimethyl sulfoxide (Sigma-Aldrich) and 0.72 μL of 5 U/μL Platinum Taq Polymerase High-Fidelity (Invitrogen), to a total volume of 25 μL. The sample plate was sealed using an ALPS 50V microplate heat sealer (Thermo Scientific). The bisulfite-treated genomic DNA template mix was then applied to a fully automated ThunderStorm system (RainDance Technologies) following the manufacturer’s instructions. In brief, primer panel droplets (MethylSeq Solution, RainDance Technologies) were dispensed to a microfluidic chip. The DNA template mix was converted into droplets within the microfluidic chip. The primer pair droplets and template droplets were then paired together in a 1:1 ratio. The paired droplets passed through an electric field inducing the discrete droplets to coalesce into a single PCR droplet (26 pL); approximately one million PCR droplets are collected per sample.

The PCR droplets were processed in a PTC-225 thermocycler (MJ Research) as follows: 94 °C for 2 min; 55 cycles of 94 °C for 30 s, 54 °C for 45 s and 68 °C for 80 s; followed by 68 °C for 10 min; 4 °C until further processing. The ramp rate was set to 1 °C per second. Following PCR amplification, 70 μL of droplet destabilizer (RainDance Technologies) were added to each sample to break the PCR droplet emulsion and release the amplicons contained within the droplets. The solution was mixed well and incubated for 15 min at room temperature. The samples were purified using Agencourt AMPure XP magnetic beads (Beckman Coulter) following the manufacturer’s protocol. For each sample, 234 μL of beads were used. The samples were eluted from magnetic beads in 40 μL of EB Buffer. The integrity and concentration (fragment range 120–300 bp) of purified amplicon DNA were assessed using a High Sensitivity DNA Kit (Agilent Technologies) on a 2100 Bioanalyzer (Agilent Technologies).

### Universal PCR

To prepare the samples for high-throughput DNA sequencing, Illumina adapter sequences and unique barcodes were introduced through an additional PCR step. Fifteen nanograms of purified amplified DNA were added to 3.25 μL of 10× High-Fidelity Buffer, 0.88 μL of 50 mM MgSO4, 0.88 μL of 10 mM dNTP solution mix, 2.50 μL of 4 mol L^−1^ betaine solution, 1.25 μL of 100% dimethyl sulfoxide, 0.50 μl of 5 U/μL Platinum Taq Polymerase High-Fidelity and 2.5 μL of 5 μM PCR primers, to a total volume of 25 μL. All primer sequences are provided in Additional file 3: Table S2.

The samples were amplified as follows: 94 °C for 2 min; 10 cycles of 94 °C for 30 s, 56 °C for 45 s and 68 °C for 60 s; followed by 68 °C for 10 min; 4 °C until further processing. DNA was purified using a MinElute PCR Purification Kit (QIAGEN) according to the manufacturer’s protocol. Purified DNA was eluted in 10 μL of EB Buffer. The samples were quantified (fragment range 100–400 bp and 220–500 bp) using a DNA 1000 Kit on a 2100 Bioanalyzer. Fifty nanograms of each sample were subsequently pooled. The resulting sequencing library was quantified using a Qubit dsDNA BR Assay Kit.

### High-throughput DNA sequencing

The pooled sequencing library (12 pmol L^−1^) and custom sequencing primers (0.5 μmol L^−1^) were applied to a MiSeq 300-cycle PE consumable cartridge (Illumina) according to the manufacturer’s protocol. The DNA sequences of the custom sequencing primers are provided in Additional file 4: Table S3. The sequencing was performed on a MiSeq DNA sequencer (Illumina) using 150 bp paired end reads.

### Data and statistical analyses

Sequencing adapters were trimmed from the raw sequencing reads using the fastq-mcf tool of ea-utils v1.1.2-537. Trimmed sequencing data were mapped to an in silico bisulfite-converted human reference genome (GRCh37) using Bismark v0.7.12 [36]. Methylation information was extracted using the methylation extractor tool of Bismark v0.7.12 [36]. Targeted DNA sequencing analyses were performed using the R package TEQC v3.2.0 [37].

## Results

### Genome-wide DNA methylation profiling to define bladder cancer-specific loci

**Fig. 1:**
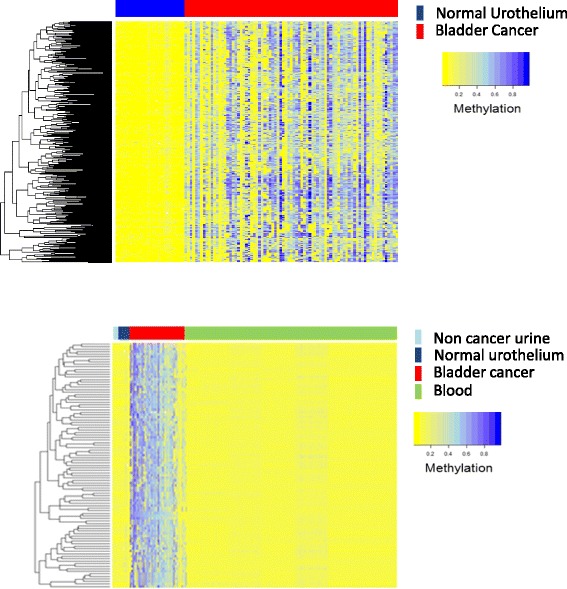
**a** Heatmap of DNA methylation state of the 432 bladder cancer-specific MVPs from the discovery cohort: tumour in *red* (*n* = 86) and normal urothelium in *blue* (*n* = 30). **b** Heatmap of the 150 loci defined in the UroMark assay: non-cancer urine in *light blue* (*n* = 10), normal urothelium in *dark blue* (*n* = 30), bladder cancer in *red* (*n* = 86) and blood in *green* (*n* = 489). The heatmap colour scale depicts methylation values ranging from 0% (*yellow*) to 100% (*blue*)

We performed genome-wide methylation profiling of 86 bladder cancers and 30 normal urothelium (Table 1) using the Infinium Human 450K DNA methylation array. From these data, we defined a panel of 432 bladder cancer-specific loci, which are unmethylated in non-cancer samples and methylated in the majority of cancer tissue (Fig. 1a).

To derive a bladder cancer-specific DNA methylation signature, which would allow classification of independent samples, we used a random forest model which resulted in a signature consisting of 150 CpG loci (Fig. 1b) which on the test set, resulted in a cross-validated sensitivity of 100% and specificity of 100% for the detection of cancer (Additional file 5: Figure S1A, B).

**Fig. 2:**
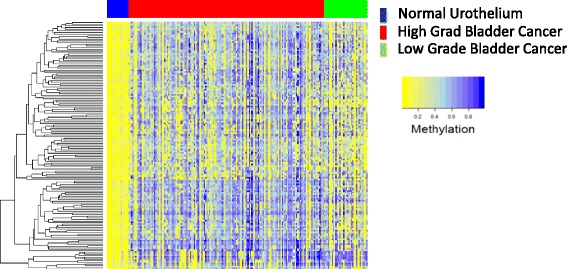
Heatmap of the 150 UroMark loci: independent primary tumours (*n* = 179) of high grade in *red* (*n* = 144) and low grade in *green* (*n* = 35); normal bladder in *blue* (*n* = 20)

Methylation data from a second cohort of 199 patients (144 high-grade muscle-invasive, 35 non-muscle-invasive cancers and 20 normal cases (Table 1, cohort 2)) was used to test the sensitivity of the marker panel for detection of bladder cancer. The panel correctly classified all bladder cancers (Fig. 2), with a resulting sensitivity and specificity of 100% (Additional file 5: Figure S2A and S2B).

### Validation of the detection panel

**Fig. 3:**
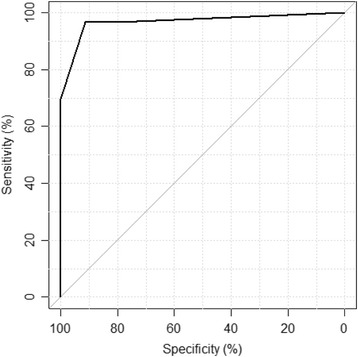
Receiver operator characteristic (ROC) evaluating the performance of the UroMark model for the detection of bladder cancer in urine

The 150 loci panel is designed for the detection of bladder cancer in urinary sediment cells. To test the assay in this setting, DNA from urinary sediment cells was extracted from a subset of 86 cases, consisting of 52 bladder cancer patients and 34 non-cancer control patients (Table 2, cohort 3). The presence of cancer was then predicted using the fixed random forest classifier defined from the discovery cohort above, and each sample was given a binary classification, cancer present/cancer absent. Figure 3 shows the receiver operator characteristics (ROC) for the UroMark assay on DNA from voided urine samples which achieved a sensitivity of 95% and specificity of 96% (AUC = 97%, negative predictive value (NPV) = 97%). Cystoscopy was used as the reference standard, and therefore, biomarker positive cystoscopy negative cases were defined as false positives.

### Developing the UroMark assay as a high-throughput NGS assay

The UroMark assay is designed as a high-throughput microdroplet-based PCR amplification system using RainDrop BS-seq [27, 28]. The encapsulation of distinct PCR reactions in microdroplets combined with NGS allows the targeted bisulfite sequencing of a large number of unique regions in parallel from limited substrate [27, 28]. We have previously validated this technology and have shown it to be highly correlated with the Infinium Human 450K DNA methylation array and also shown its utility with low template input [27, 28].

We designed a bisulfite-converted primer library to determine the methylation state of the 150 selected genomic loci. Primers were designed to interrogate both Watson and Crick strands independently where possible. Bisulfite-treated urinary DNA was subsequently used as a template for the microdroplet-based PCR amplification reaction with a RainDance ThunderStorm system.

To validate the UroMark assay using RainDrop BS-seq, we tested a second independent cohort of 188 cases (cohort 4). DNA from urinary sediment cells was obtained from 55 patients with bladder cancer, and 133 patients confirmed to be cancer-free on cystoscopy and upper track imaging (Table 2, cohort 4). All samples analysed had >30 ng of DNA; NGS data was analysed as described [27, 28]. The fraction of aligned sequencing reads mapping to the target amplicons ranged between 94.5–98.7% across the sample cohort. All amplicons of the panel amplified across the sample cohort. The average sequence coverage across all samples was 1254-fold (range 123–2673).

**Fig. 4:**
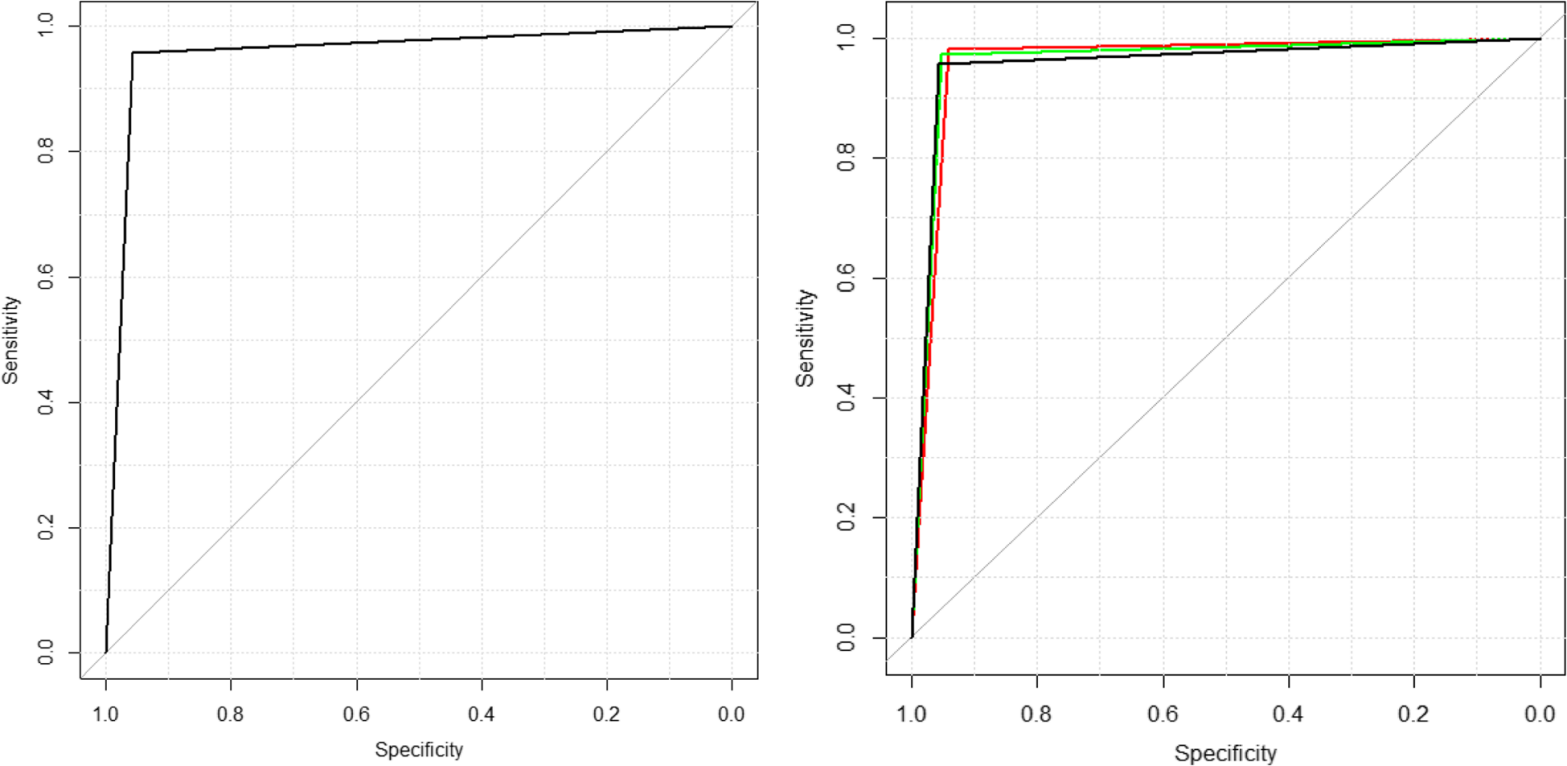
Receiver operator characteristic (ROC) evaluating the performance of the UroMark assay for the detection of bladder cancer in **a** 188 unique urine samples from cohort 4 and **b** combined total of 274 urines run through the UroMark (*red* = cohort 3, *black* = cohort 4, *green* = combined)

A methylation score for each of the 150 loci were generated using the Bismark algorithm [9]. The pre-trained fixed random forest classification model, as above, was then used to predict the presence or absence of cancer for RainDrop BS-Seq data. Using these data, the UroMark assay detected bladder cancer with a sensitivity of 96% and specificity of 97% (AUC = 96% (CI 92.66%–100); NPV = 98%) (Fig. 4a).

Combining all urine samples processed using the UroMark (*n* = 274), including non-cancer (*n* = 167) and bladder cancer (*n* = 107) (cohort 3 and cohort 4), the UroMark assay predicted the presence of bladder cancer with an AUC of 97% and a NPV of 97% (Fig. 4b).

### Comparison with small panels

To understand how the performance of the UroMark assay was compared to the best performing single markers and small-marker panels, combinations of the top performing three, five and ten biomarkers were identified from the training cohort. Any individual marker was positive based on a simple methylation cut-off generated for each locus referenced on the methylation value of normal urothelium. The best performing markers were combined into panels of three, five or ten loci along with the loci involved in previously published in regression-based predictive classifiers developed to explore the potential for these ‘oligo’ panels [19, 21–23].

These data show that although both single markers and small panels perform reasonably well alone or in combination (Table 3, Additional file 5: Figure S3), with AUCs ranging from 66–75% for small panels and 54–72% for single markers, the sensitivity to detect cancer using an oligo panel approach is limited and below a detection level desirable for clinical utility to replace cystoscopy.

**Table 3:** Test performance characteristics of small panels of markers (3, 5 and 10) and single markers to detect bladder cancer compared to the UroMark assay

UroMark	UroMark	Specificity	Sensitivity	NPV	PPV	AUC
		1	0.98	0.97	1	0.9829
Top panels	Top 10	0.91	0.91	0.72	0.94	0.7525
	Top 5	0.89	0.77	0.66	0.91	0.6993
	Top 3	0.86	0.72	0.61	0.88	0.6604
Top individual marker	Marker_1	0.89	0.62	0.54	0.88	0.6173
	Marker_2	0.91	0.75	0.67	0.93	0.7197
	Marker_3	0.83	0.73	0.59	0.85	0.6319
	Marker_4	0.89	0.84	0.73	0.91	0.7321
	Marker_5	0.91	0.57	0.53	0.91	0.6213
	Marker_6	0.94	0.59	0.54	0.94	0.6438
	Marker_7	0.86	0.79	0.67	0.90	0.6932
	Marker_8	0.74	0.81	0.66	0.83	0.6382
	Marker_9	0.86	0.51	0.47	0.83	0.5457
	Marker_10	0.94	0.69	0.61	0.95	0.693

## Discussion

This study reports the development and proof of principle testing of high-throughput target bisulphite sequencing assay, UroMark, to interrogate cancer epigenetic alterations in urinary sediment. A non-invasive test for the detection of bladder cancer has the potential to revolutionise the diagnostic pathway, for both haematuria investigations and improved surveillance strategies for patients with established disease. In proof of concept testing of a cohort of cancer and control urine samples, we show that the high sensitivity and specificity obtained with the UroMark assay has performance characteristics which are similar to cystoscopy.

DNA methylation patterns are highly cancer cell-specific, and the ontogenic stability of these epigenetic events makes DNA methylation an ideal biomarker for the detection and diagnosis of disease. Changes in global DNA methylation patterns are a common feature of neoplastic transformation and a frequent event in bladder cancer [16–18, 38]. Previous studies have shown that changes in DNA methylation status of bladder cancer, both non-muscle-invasive and muscle-invasive bladder cancers as well as normal urothelium are reflected in the methylation status of urinary sediment cells and as such, could be a useful diagnostic marker [16, 17, 38]. Although methylation-based detection assays (alone or in combination with somatic mutation) show promise with sensitivity to detect bladder cancer between 65–98%, they have not as yet progressed into clinical practise [8, 17, 19, 21–24]. The number of loci that can be included within a panel using traditional technology to detect methylation and the low volume of substrate DNA that can be extracted from urinary sediment for large numbers of candidates have until recently been a limiting factor for assay development. Furthermore, the reliance on uniform methylation (low inter-tumour variability) as well as the effect of intra-tumour heterogeneity indicate that the performance of ‘oligo panel’ assays will be limited across a wide spectrum of stage and grade [39]. Novel technologies, combining next-generation bisulfite sequencing with large-scale multiplex PCR, overcome these issues allowing the interrogation of a large panel of epigenetic biomarkers from a single sample, and as such, we believe this represents a paradigm shift in development strategy [27, 28].

In order to annotate the epigenetic alterations involved in bladder cancer and to define a biomarker panel, we have carried out the largest unbiased genome-wide DNA methylation screens of bladder cancer to date. Although our initial discovery panel was predominately high-grade disease, it is of note that the majority or >98% of the alterations present in high-grade bladder cancer were confirmed in low-grade disease. Using stringent criteria, we have defined a detection panel of 150 CpG loci which is relatively large but was necessary to detect all of the 260 bladder cancers included in the assay development cohort based on individual methylation expression and representing the spectrum of stage and grade. We believe the large panel and customised random forest analysis pipeline to retain specificity and sensitivity can overcome shortcomings of traditional biomarker panels which are constrained by technology. The use of NGS, particularly in cancer diagnosis, is becoming routine, and consistent with this, we demonstrate the potential for a large-scale highly multiplexed next-generation assay, the performance characteristics being highly sensitive and specific, can be achieved with a NPV comparable to cystoscopy.

The current assay was developed to answer the specific question around the primary diagnosis of bladder cancer in patients with haematuria and has yet to be tested in the recurrence setting. Urinary biomarker assays for the detection of recurrent bladder cancer have generally fared less well than in the primary diagnosis setting with sensitivities ranging from 56–80%. This low sensitivity for the detection of recurrent disease is likely due to low urinary concentration of tumour cells harbouring the small number specific alterations analysed or the analytical sensitivity of the assays used. The large panel of loci utilised in the UroMark panel in combination with the analytical sensitivity potential achieved with next generation sequencing may also allow the UroMark assay to compare favourably with cystoscopy in the recurrent setting.

The next stage will be the robust testing (Phase III biomarker road map [40]) of this assay in two MRC-funded trials (NCT02676180 and NCT0278428) which are currently recruiting across multiple sites in the UK. In the current development studies, we used a mixed cohort of tumours and non-cancer controls from various sources. The objective of the Phase III studies is to determine the NPV of UroMark for the detection of bladder cancer in a population of patients referred for investigation of haematuria.

## Conclusions

In this proof of concept study, we show the potential utility of a highly multiplex bisulphite sequencing assay for the detection of bladder cancer from urinary sediment. The use of a non-invasive assay which rules out the presence of cancer with a high degree of certainty has the potential to revolutionise the treatment of bladder cancer.

## Additional files

Additional file 1: Table S4. Test performance of individual loci. (XLSX 14 kb)
Additional file 2: Table S1. RainDance target primers. (XLSX 13 kb)
Additional file 3: Table S2.﻿ RainDance barcode primer sequences. (XLSX 12 kb)
Additional file 4: Table S3. RainDance custom sequencing primers. (XLSX 8 kb)
Additional file 5: Figure S1. A) MDS plot of 150 UroMark loci panel, tumour = red, normal = blue, B) ROC for cross validation accuracy of 150 loci UroMark assay. **Figure S2.** A) MDS plot of 150 UroMark loci panel in 179 sample validation cohort, High grade = red, low grade = green, normal = blue, B) ROC for cross validation accuracy of 150 loci UroMark assay in the 179 sample validation cohort. **Figure S3.** A) Boxplots of methylation values for top 10 performing markers in the primary tissue training cohort. B) Boxplots of methylation values for top 10 performing markers in the in urine samples validation cohorts (normal – confirmed no tumour, tumour - histological confirmation of TCC). **Figure S4.** A) Boxplots of methylation values for top 10 performing markers in the primary tissue training cohort. B) Boxplots of methylation values for top 10 performing markers in the in urine samples validation cohorts (normal – confirmed no tumour, tumour histological confirmation of TCC). (PDF 164 kb)
