# Supplementary material for: UroMark—a urinary biomarker assay for the detection of bladder cancer
Source: Clin Epigenetics. 2017 Jan 31;9:8. doi: 10.1186/s13148-016-0303-5 (PMC5282868; doi:10.1186/s13148-016-0303-5)
Supplement: Additional file 5: Figure S1. — A) MDS plot of 150 UroMark loci panel, tumour = red, normal = blue, B) ROC for cross validation accuracy of 150 loci UroMark assay. Figure S2. A) MDS plot of 150 UroMark loci panel in 179 sample validation cohort, High grade = red, low grade = green, normal = blue, B) ROC for cross validation accuracy of 150 loci UroMark assay in the 179 sample validation cohort. Figure S3. A) Boxplots of methylation values for top 10 performing markers in the primary tissue training cohort. B) Boxplots of methylation values for top 10 performing markers in the in urine samples validation cohorts (normal – confirmed no tumour, tumour - histological confirmation of TCC). Figure S4. A) Boxplots of methylation values for top 10 performing markers in the primary tissue training cohort. B) Boxplots of methylation values for top 10 performing markers in the in urine samples validation cohorts (normal – confirmed no tumour, tumour histological confirmation of TCC). (PDF 164 kb) [file 13148_2016_303_MOESM5_ESM.pdf]

Supplementary Figure 1

A) B)

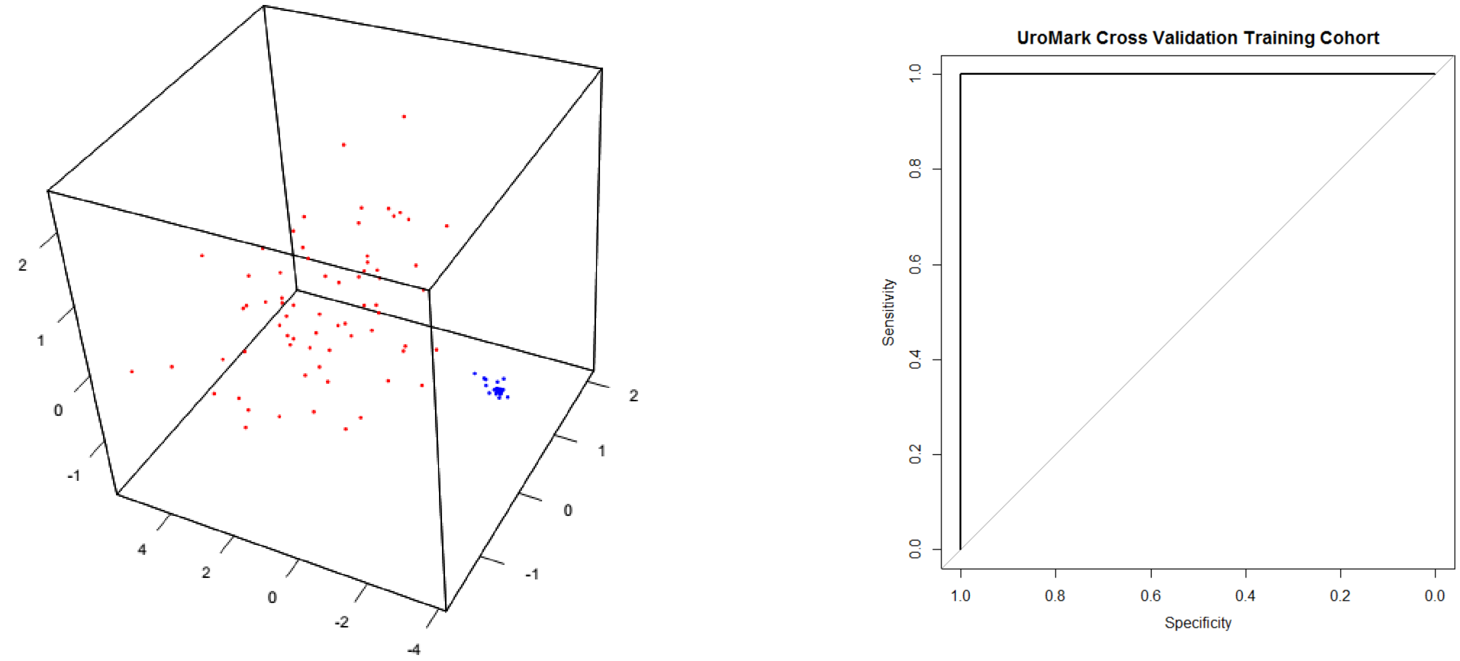

Supplementary Figure 1 A) MDS plot of 150 UroMark loci panel, tumour = red, normal = blue, B) ROC for cross validation accuracy of 150 loci UroMark assay.

Supplementary Figure 2

A) B)

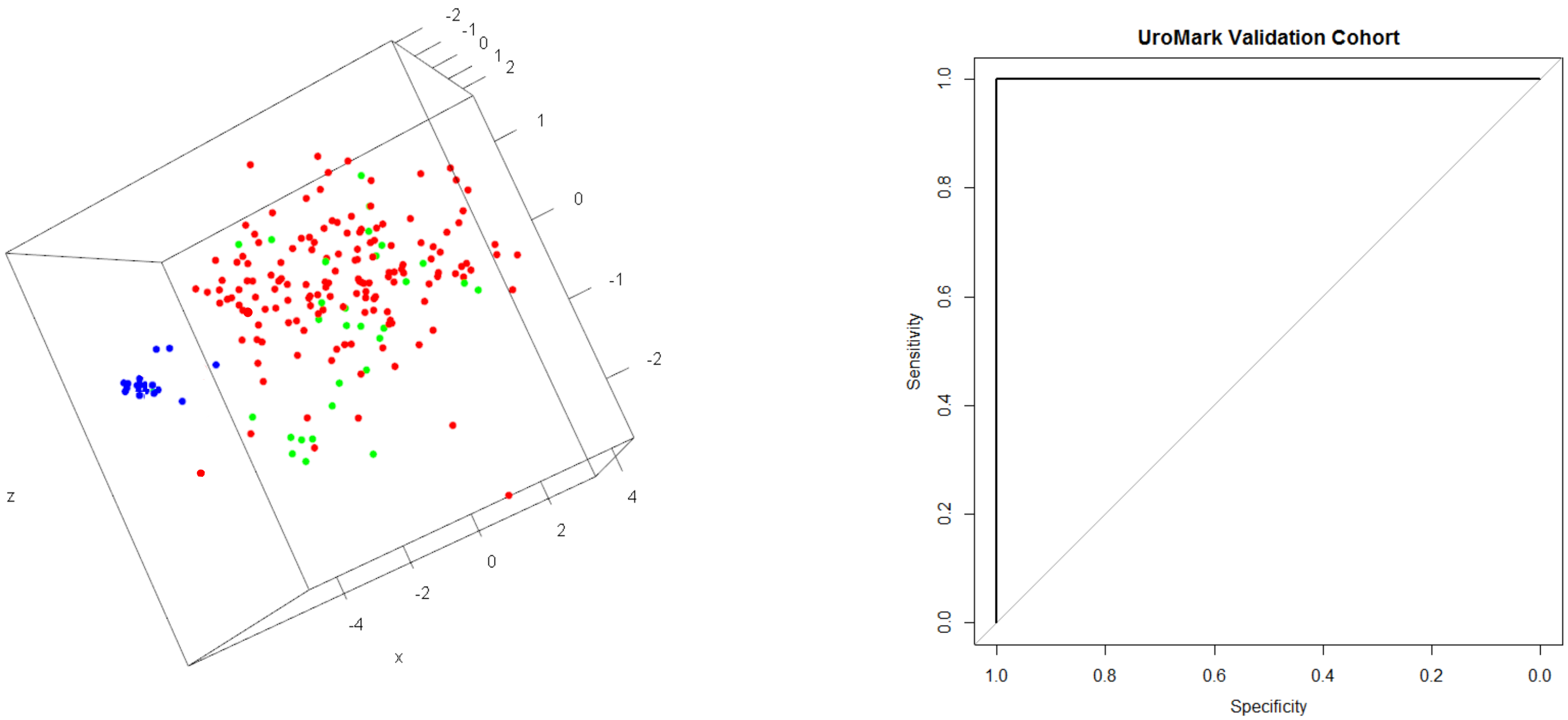

Supplementary Figure 2 A) MDS plot of 150 UroMark loci panel in 179 sample validation cohort, High grade = red, low grade = green, normal = blue, B) ROC for cross validation accuracy of 150 loci UroMark assay in the 179 sample validation cohort.

Supplementary Figure 3

A)

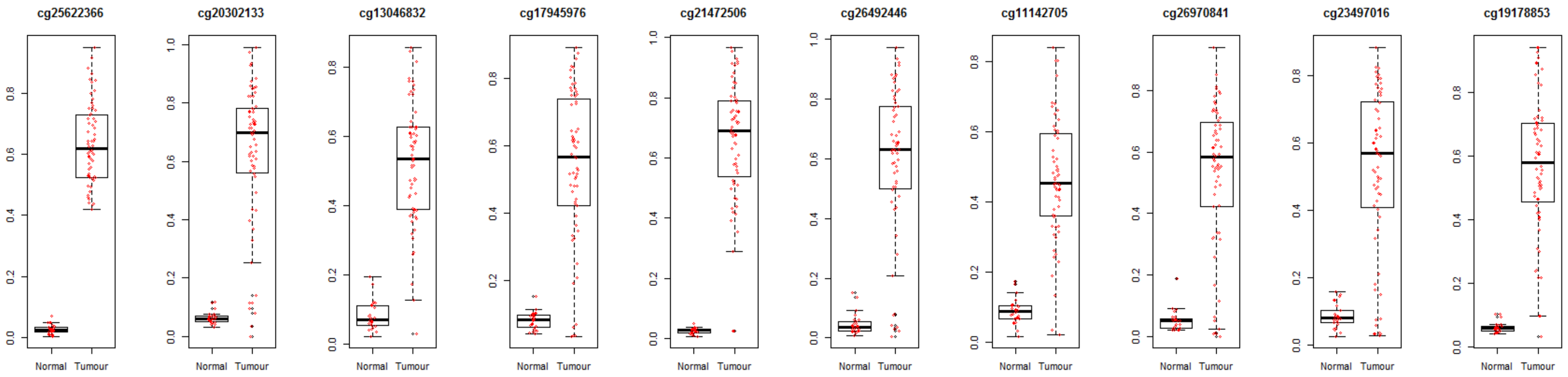

B)

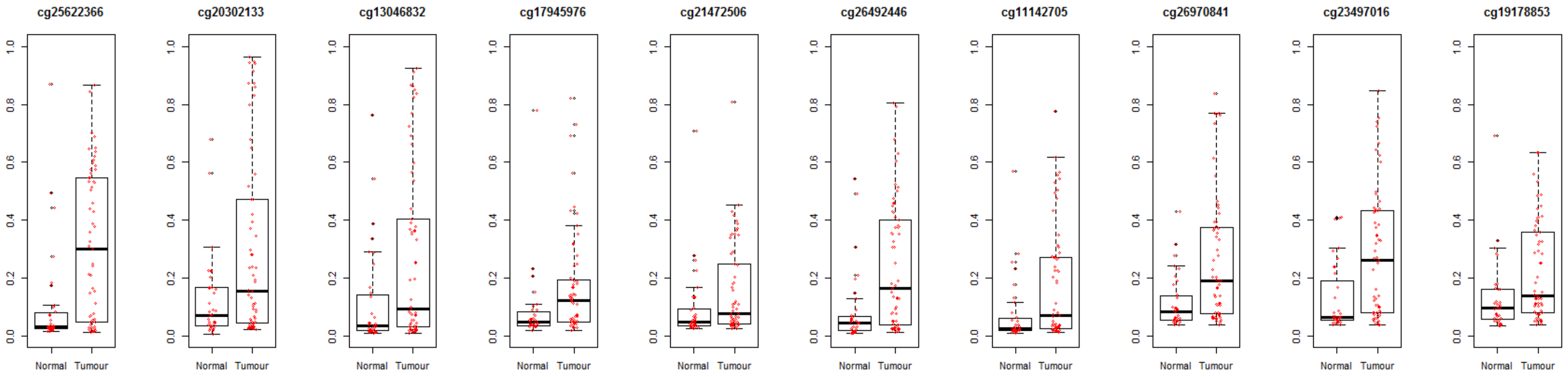

Supplementary Figure 3 A) Boxplots of methylation values for top 10 performing markers in the primary tissue training cohort. B) Boxplots of methylation values for top 10 performing markers in the in urine samples validation cohorts (normal – confirmed no tumour, tumour - histological confirmation of TCC) .

Supplementary Figure 4

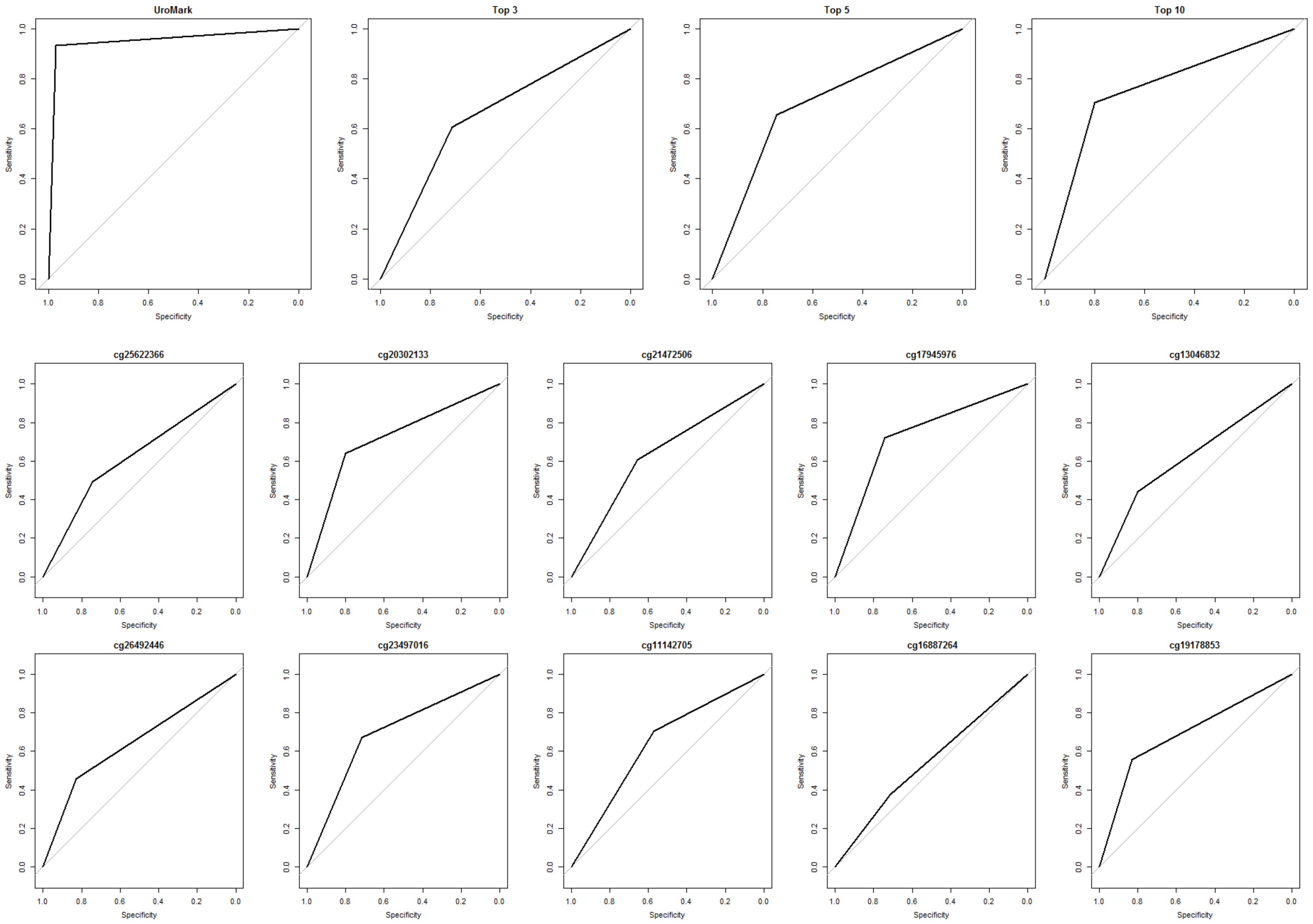

Supplementary Figure 4 A) Boxplots of methylation values for top 10 performing markers in the primary tissue training cohort. B) Boxplots of methylation values for top 10 performing markers in the in urine samples validation cohorts (normal – confirmed no tumour, tumour histological confirmation of TCC) .
